# On Preserved Meat-Juice

**Published:** 1855-03

**Authors:** Robert Chrisitson

**Affiliations:** Prof. of Materia Medica in the University of Edinburgh


*On Preserved Meat-Juice.*—By ROBERT CHRISITSON, M. D., Prof. of Materia Medica in the University of Edinburgh.—About 18 months ago, when consulted in the case of a relative of Mr. Gillon, the extensive and skilful manufacturer of preserved meats at Leith, I found that the patient was entirely supported in a severe illness, by the Preserved Juice of Meat, which had been given at Mr Gillon's suggestion. Observing the readiness with which it was taken when other food of every kind was refused, I was induced to try it in other instances, and eventually to employ it in various states of disease. The results led me to suggest the use of it to many professional friends, and to advise the druggists of Edinburgh to keep it, so that it is now much in request, and may be easily obtained.

This substance is the pure juice of beef, preserved in the way in which meats and vegetables are now so extensively preserved in the fresh state, for store provisions. The mode of preparation is as follows: Cylindrical cases of tinned iron are filled each with six pounds and a half of beef; and the lid is soldered on, but with a hole about half an inch in diameter in the middle of it. Two trays of such cases are shoved into iron retorts, analogous in form to retorts for gas-making, but double-cased, so that steam may be introduced into the interstice around. They are thus subjected to a heat of 220° under steam pressure, for about three hours; by which the beef is partially cooked, and, being thus also made to contract strongly on itself, squeezes out a portion of its juice, amounting to a few ounces from each tin. The tins are then drawn, the juice is poured out, and the meat, with certain additions, is subjected to the preservative process. The juice, after being cooled and entirely freed from fat, is put into small four-ounce tin cases. Each of these has a small aperture at one end, which is secured by solder, after the juice is poured in. The tins are then subjected, on trays, to a temperature of 220° in a muriate of lime bath. On being removed, the solderer rapidly touches with his iron the solder on top, which giving way allows steam to rush out forcibly, and carry with it the air in the upper part of the interior. By the time he has thus swiftly passed over sixteen or twenty tins, the first is ready for being re-soldered by a similar dexterous application of his iron, which then in succession as quickly secures the whole open and steaming apertures. The process of heating in the bath, tapping and resoldering, is then repeated a second time, to make sure of the thorough expulsion of every particle of air. The tins finally are painted to preserve them against rust.

The process is most perfect. I have repeatedly opened tins eighteen months in my possession, and stated to have been many months in store when I got them, and in every instance the contents had the rich delicate aroma and taste of fresh beef-juice. Sometimes the taste is slightly resinous or soapy, in consequence of a little resin having obtained admission in the operation of soldering. But as this does not occur often, the impurity may be avoided with due care. The juice may be taken with relish in small quantity, either cold or warm, in its concentrated shape; but it is rather strong to be used without dilution. When diluted with three times its volume of boiling water, and duly seasoned with salt and pepper, it makes a more palatable beef-tea than any which can be made in the usual way. Sometimes, indeed, a patient will be found to prefer the ordinary sort, either because the preserved juice has unluckily been resinous, or on the same principle that leads some people from the plains of England to prefer hard water to the pure mountain springs of the primitive districts of Scotland, viz., because they are not accustomed to the finer sort. But this is not the general fact; and there can be no doubt that the preserved meat-juice makes a most palatable beef-tea, and an equally eligible basis for many soups.

Until about ten years ago, in concurrence with general opinion, I used to regard beef tea as a highly nutritive article, not to be rashly or freely given during disease. My sentiments in this respect were shaken, when I ascertained, in the course of some experiments for adjusting the dietaries of the General Prison and the Royal Infirmary, that a pint of the very finest beef tea contained scarcely a quater of an ounce of anything but water. Since that time I have much more readily listened to the cravings of patients for beef-tea in even many acute diseases, and above all in protracted sub-acute diseases, and in chronic diseases with fever; and I have thought I saw that it maintains the strength almost like wine, lessons emaciation and weakness in tedious diseases, and does not occasion any increase of reaction. There is no disease in which these properties are more remarkably shown than in protracted cases of gastric fever, of which by the way, I have seen an unusual number, both in town and country, during the last three years. These cases have often lasted for six weeks, or,—with a relapse, from too early indulgence or exposure,—for the long term of three months nearly: during which little, or absolutely nothing else, was taken, except beef-tea or diluted meat-juice; and without the attenuation and debility which so protracted a fever and want of appetite ought to have induced. In some instances I could scarcely doubt that life was preserved by this nutriment. It is unnecessary to particularise the various states of diseases in which the same practice has been followed. It is peculiarly applicable to all subacute protracted diseases, whether febrile or otherwise; and in all such there is even no great reason to hesitate in resorting to it when local inflammation is present. Every one, I think, will be struck with the readiness with which such patients will often take diluted meat-juice or beef-tea repeatedly, when they refuse all other kinds of food. It should be given in the quantity of a teacupful at a time, every four or six hours; but it is well to alternate it with other simple nourishment, when the patient will consent to do so.

What is its mode of action? Not simply nutrient. A quarter of an ounce of the most nutritive material cannot nearly replace the daily wear and tear of the tissues in any circumstances. Possibly it belongs to a new denomination of remedies, whose action never was even suspected to exist until recently—those which, by some peculiar influence, diminish the waste of the tissues under the exercise of their functions. Professor Lehmann has proved (*Annalen der Chemie*, 1853) that coffee possesses this singular property in so remarkable a degree, that in persons following an active occupation an infusion of an ounce of roasted coffee daily will reduce the daily waste by a fourth part; and the same property seems likewise to belong to tea, and other restorative beverages, It is not improbable that the sapid and saline principles of meat, united in what is called ozmazome, and constituting the ingredients of beef-tea and meat-juice, possess some such property. It is difficult otherwise to account for the interesting results obtained by the late Dr. Edwards, in 1833, who, in his researches on nutrition,—strangely overlooked by the celebrated Gelatin Commission of the French Institute, in their condemnatory report on gelatin in 1841,—found that dogs die slowly if fed on bread and gelatin alone, but, when thus greatly reduced, quickly regain flesh and strength by the addition of two ounces of meat-tea, which cannot appreciably increase their textures by its own insignificant amount of solids. Either it acts as a digestive ferment, so to speak,—promoting the assimilation of other nutriment—or, like coffee, it must lessen the waste of the tissues in the exercise of their functions.

Mr. Gillon's meat-juice contains only 6½ per cent. of solids. As a mere nutrient, therefore, it is much in the same cetegory with beef-tea. Sixteen ounces of beef-tea, made with the contents of one tin, yield only 114 grains of solid extract. It contains no fibrin, no albumen, no gelatin. It does not even gelatinise, on exposure to the air for days: it is ozmazome, with the salts and sapid and odorous principles of meat, and materially different from all boiled extracts.

I should add that no good beef-tea could be made so cheap as with this preserved meat-juice. A tin of four ounces makes sixteen of strong beef-tea. This much requires, in the ordinary way, a pound of the finest beef, which at present costs ninepence, and is scarcely ever so cheap as sixpence. The reason for the cheapness of Mr. Gillon's meat-juice is, that the residual meat is economised, while that of the ordinary cooking process is good for nothing.

It is a much more convenient article for use than any of the extracts made from meat by extemporary processes in the kitchen, or by certain very dubious chemical methods lately come into vogue. It differs materially from all meat extracts prepared by boiling.—*London Monthly Journal of Medicine.*

